# Current Mathematical Methods Used in QSAR/QSPR Studies

**DOI:** 10.3390/ijms10051978

**Published:** 2009-04-29

**Authors:** Peixun Liu, Wei Long

**Affiliations:** Institute of Radiation Medicine, Peking Union Medical College, Chinese Academy of Medical Sciences, Tianjin 300192, P.R. China; E-Mails: longwaylong@gmail.com (W.L.); pharm8888@yahoo.com.cn (P.-X.L.); Tel. +86-22-85683042; Fax: +86-22-85682394

**Keywords:** QSAR, QSPR, Mathematical methods, Regression, Algorithm

## Abstract

This paper gives an overview of the mathematical methods currently used in quantitative structure-activity/property relationship (QASR/QSPR) studies. Recently, the mathematical methods applied to the regression of QASR/QSPR models are developing very fast, and new methods, such as Gene Expression Programming (GEP), Project Pursuit Regression (PPR) and Local Lazy Regression (LLR) have appeared on the QASR/QSPR stage. At the same time, the earlier methods, including Multiple Linear Regression (MLR), Partial Least Squares (PLS), Neural Networks (NN), Support Vector Machine (SVM) and so on, are being upgraded to improve their performance in QASR/QSPR studies. These new and upgraded methods and algorithms are described in detail, and their advantages and disadvantages are evaluated and discussed, to show their application potential in QASR/QSPR studies in the future.

## 1. Introduction

As a common and successful research approach, quantitative structure activity/property relationship (QASR/QSPR) studies are applied extensively to chemometrics, pharmacodynamics, pharmacokinetics, toxicology and so on. Recently, the mathematical methods used as regression tools in QSAR/QSPR analysis have been developing quickly. Thus, not only are the previous methods, such as Multiple Linear Regression (MLR), Partial Least Squares (PLS), Neural Networks (NN), Support Vector Machine (SVM), being upgraded by improving the kernel algorithms or by combining them with other methods, but also some new methods, including Gene Expression Programming (GEP), Project Pursuit Regression (PPR) and Local Lazy Regression (LLR), are being mentioned in the current reported QSAR/QSPR studies. In light of this, this paper is divided as follows: first, the improved methods based on the earlier ones are described, then the new methods are presented. Finally, all these methods are jointly commented and their advantages and disadvantages discussed to show their application potential in future QASR/QSPR studies.

## 2. Multiple Linear Regression (MLR)

MLR is one of the earliest methods used for constructing QSAR/QSPR models, but it is still one of the most commonly used ones to date. The advantage of MLR is its simple form and easily interpretable mathematical expression. Although utilized to great effect, MLR is vulnerable to descriptors which are correlated to one another, making it incapable of deciding which correlated sets may be more significant to the model. Some new methodologies based on MLR have been developed and reported in recent papers aimed at improving this technique. These methods include Best Multiple Linear Regression (BMLR), Heuristic Method (HM), Genetic Algorithm based Multiple Linear Regression (GA-MLR), Stepwise MLR, Factor Analysis MLR and so on. The three most important and commonly used of these methods are described in detail below.

### 2.1. Best Multiple Linear Regression (BMLR)

BMLR implements the following strategy to search for the multi-parameter regression with the maximum predicting ability. All orthogonal pairs of descriptors i and j (with R2ij < R2min, default value R2ij < 0.1) are found in a given data set. The property analyzed is treated by using the two-parameter regression with the pairs of descriptors, obtained in the first step. The Nc (default value Nc = 400) pairs with highest regression correlation coefficients are chosen for performing the higher-order regression treatments. For each descriptor pair, obtained in the previous step, a non-collinear descriptor scale, k (with R2ik < R2nc and R2kj < R2nc, default value R2 < 0.6) is added, and the respective three-parameter regression treatment is performed. If the Fisher criterion at a given probability level, F, is smaller than that for the best two-parameter correlation, the latter is chosen as the final result. Otherwise, the Nc (default value Nc = 400) descriptor triples with highest regression correlation coefficients are chosen for the next step. For each descriptor set, chosen in the previous step, an additional non-collinear descriptor scale is added, and the respective *(n + 1)*-parameter regression treatment is performed. If the Fisher criterion at the given probability level, *F*, is smaller than for the best two-parameter correlation, the latter is chosen as the final result. Otherwise, the *Nc* (default value *Nc =* 400) sets descriptor sets with highest regression correlation coefficients are chosen, and this step repeated with *n = n + 1* [1].

As an improved method based on MLR, BMLR is instrumental for variable selection and QSAR/QSPR modeling [2–8]. Like MLR, BMLR is noted for its simple and interpretable mathematical expression. Moreover, overcoming the shortcomings of MLR, BMLR works well when the number of compounds in the training set doesn’t exceed the number of molecular descriptors by at least a factor of five. However, BMLR will derive an unsatisfactory result when the structure-activity relationship is non-linear in nature. When too many descriptors are involved in a calculation, the modeling process will be time consuming. To speed up the calculations, it is advisable reject descriptors with insignificant variance within the dataset. This will significantly decrease the probability of including unrelated descriptors by chance. In addition, BMLR is unable to build a one-parameter model. BMLR is commercially available in the software packages CODESSA [9] or CODESSA PRO [10].

### 2.2. Heuristic Method (HM)

HM, an advanced algorithm based on MLR, is popular for building linear QSAR/QSPR equations because of its convenience and high calculation speed. The advantage of HM is totally based on its unique strategy of selecting variables. The details of selecting descriptors are as follows: first of all, all descriptors are checked to ensure that values of each descriptor are available for each structure. Descriptors for which values are not available for every structure in the data are discarded. Descriptors having a constant value for all structures in the data set are also discarded. Thereafter all possible one-parameter regression models are tested and the insignificant descriptors are removed. As a next step, the program calculates the pair correlation matrix of descriptors and further reduces the descriptor pool by eliminating highly correlated descriptors. The details of validating intercorrelation are: (a) all quasi-orthogonal pairs of structural descriptors are selected from the initial set. Two descriptors are considered orthogonal if their intercorrelation coefficient *r_ij_* is lower than 0.1; (b) the pairs of orthogonal descriptors are used to compute the biparametric regression equations; (c) to a multi-linear regression (MLR) model containing *n* descriptors, a new descriptor is added to generate a model with *n + 1* descriptors if the new descriptor is not significantly correlated with the previous *n* descriptors; step (c) is repeated until MLR models with a prescribed number of descriptors are obtained. The goodness of the correlation is tested by the square of coefficient regression (*R^2^*), square of cross-validate coefficient regression (*q^2^*), the F-test (*F*), and the standard deviation (*S*) [1].

HM is commonly used in linear QSAR and QSPR studies, and also as an excellent tool for descriptor selection before a linear or nonlinear model is built [11–34]. The advantages of HM are the high speed and the absence of software restrictions on the size of the data set. HM can either quickly give a good estimation about what quality of correlation to expect from the data, or derive several best regression models. HM usually produces correlations 2 – 5 times faster than other methods with comparable quality. Additionally, the maximum number of parameters in the resulting model can be fixed in accordance with the situation so as to save time. As a method inherited from MLR, HM is also limited in linear models.

### 2.3. Genetic Algorithm based Multiple Linear Regression (GA-MLR)

Combining Genetic Algorithm (GA) with MLR, a new method called GA-MLR is becoming popular in currently reported QSAR and QSPR studies [35–55]. In this method, GA is performed to search the feature space and select the major descriptors relevant to the activities or properties of the compounds. This method can deal with z large search space efficiently and has less chance to become a local optimal solution than the other algorithms. We give a brief summary of the main procedure of GA herein. The first step of GA is to generate a set of solutions (chromosomes) randomly, which is called an initial population. Then, a fitness function is deduced from the gene composition of a chromosome. The Friedman LOF function is commonly used as the fitness function, which was defined as follows:

(1) $$\text{LOF}={\left\{\text{SSE}/(1-(c+dp/n))\right\}}^{2}$$

where SSE is the sum of squares of errors, *c* is the number of the basis function (other than the constant term), *d* is the smoothness factor, *p* is the number of features in the model, and *n* is the number of data points from which the model is built. Unlike the *R^2^* error, the LOF measure cannot always be reduced by adding more terms to the regression model. By limiting the tendency to simply add more terms, the LOF measure resists over-fitting of a model. Then, crossover and mutation operations are performed to generate new individuals. In the subsequent selection stage, the fittest individuals evolve to the next generation. These steps of evolution continue until the stopping conditions are satisfied. After that, the MLR is employed to correlate the descriptors selected by GA and the values of activities or properties.

GA, a well-estimated method for parameter selection, is embedded in GA-MLR method so as to overcome the shortage of MLR in variable selection. Like the MLR method, the regression tool in GA-MLR, is a simple and classical regression method, which can provide explicit equations. The two parts have a complementation for each other to make GA-MLR a promising method in QSAR/QSPR research.

## 3. Partial Least Squares (PLS)

The basic concept of PLS regression was originally developed by Wold [56,57]. As a popular and pragmatic methodology, PLS is used extensively in various fields. In the field of QSAR/QSPR, PLS is famous for its application to CoMFA and CoMSIA. Recently, PLS has evolved by combination with other mathematical methods to give better performance in QSAR/QSPR analyses. These evolved PLS’, such as Genetic Partial Least Squares (G/PLS), Factor Analysis Partial Least Squares (FA-PLS) and Orthogonal Signal Correction Partial Least Squares (OSC-PLS), are briefly introduced in the following sections.

### 3.1. Genetic Partial Least Squares (G/PLS)

G/PLS is derived from two QSAR calculation methods Genetic Function Approximation (GFA) [58,59] and PLS. The G/PLS algorithm uses GFA to select appropriate basis functions to be used in a model of the data and PLS regression is used as the fitting technique to weigh the basis functions’ relative contributions in the final model. Application of G/PLS thus allows the construction of larger QSAR equations while still avoiding over-fitting and eliminating most variables. As the regression method used in Molecular Field Analysis (MFA), a well-known 3D-QSAR analysis tool, G/PLS is commonly used. The recent literatures related to G/PLS are mainly listed as [60–70].

### 3.2. Factor Analysis Partial Least Squares (FA-PLS)

This is the combination of Factor Analysis (FA) and PLS, where FA is used for initial selection of descriptors, after which PLS is performed. FA is a tool to find out the relationships among variables. It reduces variables into few latent factors from which important variables are selected for PLS regression. Most of the time, a leave-one-out method is used as a tool for selection of optimum number of components for PLS. We can find examples of FA-PLS used in QSAR analysis in [68,71–74].

### 3.3. Orthogonal Signal Correction Partial Least Squares (OSC-PLS)

Orthogonal signal correction (OSC) was introduced by Wold *et al.* [75] to remove systematic variation from the response matrix *X* that is unrelated, or orthogonal, to the property matrix *Y*. Therefore, one can be certain that important information regarding the analyte is retained. Since then, various OSC algorithms have been published in an attempt to reduce model complexity by removing orthogonal components from the signal. *In abstracto*, a preprocessing with OSC will help traditional PLS to obtain a more precise model, as proven in many studies of spectral analysis [76–88]. To date, unfortunately, there are only a few reports in which OSC-PLS is applied to QSAR/QSPR studies [89–91], but more QSAR or QSPR research involving application of the OSC-PLS method are expected in the future.

## 4. Neural Networks (NN)

As an alternative to the fitting of data to an equation and reporting the coefficients derived therefrom, neural networks are designed to process input information and generate hidden models of the relationships. One advantage of neural networks is that they are naturally capable of modeling nonlinear systems. Disadvantages include a tendency to overfit the data, and a significant level of difficulty in ascertaining which descriptors are most significant in the resulting model. In the recent QSAR/QSPR studies, RBFNN and GRNN are the most frequently used ones among NN.

### 4.1. Radial Basis Function Neural Network (RBFNN)

The RBFNN consists of three layers: an input layer, a hidden layer and an output layer. The input layer does not process the information; it only distributes the input vectors to the hidden layer. Each neuron on the hidden layer employs a radial basis function as a nonlinear transfer function to operate on the input data. In general, there are several radial basis functions (RBF): linear, cubic, thin plate spline, Gaussian, multi-quadratic and inverse multi-quadratic. The most often used RBF is a Gaussian function that is characterized by a center (*c_j_*) and width (*r_j_*). Due to the limited length of writing, only Gaussian RBF is introduced in this paper. The nonlinear transformation with RBF in the hidden layer is given as follows:

(2) $$h_{j}(x)=\text{exp}\left(\frac{-{\left\|x-c_{j}\right\|}^{2}}{r_{j}^{2}}\right)$$

where, *h_j_* is the notation for the output of the *j*th RBF unit, *c_j_* and *r_j_* are the center and width of the *j*th RBF, respectively. The operation of the output layer is linear, which is given as below:

(3) $$y_{k}(x)=\sum w_{kj}h_{j}(x)+b_{k}$$

where, *y_k_* is the *k*th output unit for the input vector *x*, *w_kj_* is the weight connection between the *k*th output unit and the *j*th hidden layer unit, and *b_k_* is the bias. The training procedure when using RBF involves selecting centers, width and weights. In this paper, the forward subset selection routine was used to select the centers from training set samples. The adjustment of the connection weight between the hidden layer and the output layer was performed using a least squares solution after the selection of centers and widths of radial basis functions. Compared with the Back Propagation Neural Network (BPNN), RBFNN has the advantage of short training time and is guaranteed to reach the global minimum of error surface during training process. The optimization of its topology and learning parameters are easy to implement. Applications of RBFNN in QSAR/QSPR studies can be found in [22,24,27,32,51,92–102].

### 4.2. General Regression Neural Network (GRNN)

GRNN, one of the so-called Bayesian networks, is a type of neural network using kernel-based approximation to perform regression. It was introduced by Specht in 1991 [103]. GRNN is a nonparametric estimator that calculates a weighted average of the target values of training patterns by the probability density function using Parzen’s nonparametric estimator. For GRNN, the predicted value is the most probable value *E*(*y*|*x*):

(4) $$E(y|x)\overset{□}{=}\hat{y}(x)=\frac{\int_{-\infty }^{+\infty }yf\;(x,y)\text{dy}}{\int_{-\infty }^{+\infty }f\;(x,y)\text{dy}}$$

where *f*(*x, y*) is the probability density function. This can be estimated from the training set by using the Parzen’s nonparametric estimator [47]:

(5) $$f(x,y)=\frac{1}{n\sigma }\sum_{i=1}^{n}w\frac{\left(x-x_{i}\right)}{\sigma }$$

where *n* is the sample size, s is a scaling parameter that defines the width of the bell curve that surrounds each sample point, *W*(*d*) is a weighting function that has its largest value at *d*=0, and (*x-x_i_*) is the distance between the unknown sample and a data point. The Gaussian function is frequently used as the weighting function because it is well behaved, easily calculated, and satisfies the conditions required by Parzen’s estimator. Substituting Parzen’s nonparametric estimator for *f*(*x, y*) and performing the integrations leads to the fundamental equation of GRNN:

(6) $$\hat{y}(x)=\frac{\sum_{i=1}^{n}y_{i}\;\text{exp}(-D(x,x_{i}))}{\sum_{i=1}^{n}\text{exp}(-D(x,x_{i}))}$$

where:

(7) $$D(x,x_{i})=\sum_{j=1}^{p}{\left(\frac{x_{j}-x_{ij}}{\sigma_{j}}\right)}^{2}$$

GRNN consists of four layers: input, hidden, summation, and output layers. The greatest advantage of GRNN is the training speed. Meanwhile, it is relatively insensitive to outliers (wild points). Training a GRNN actually consists mostly of copying training cases into the network, and so is as close to instantaneous as can be expected. The greatest disadvantage is network size: a GRNN network actually contains the entire set of training cases, and is therefore space-consuming, requiring more memory space to store the model. Relative literatures are [46,104–109].

## 5. Support Vector Machine (SVM)

SVM, developed by Vapnik [110,111] as a novel type of machine learning method, is gaining popularity due to its many attractive features and promising empirical performance. Originally, SVM was developed for pattern recognition problems. After that, SVM it was applied to regression by the introduction of an alternative loss function and results appear to be very encouraging [112]. As a developing method, new types of SVM are coming in on the stage of QSAR/QSPR, such as Least Square Support Vector Machine (LS-SVM), Grid Search Support Vector Machine (GS-SVM), Potential Support Vector Machine (P-SVM) and Genetic Algorithms Support Vector Machine (GA-SVM). Here, we only choose LS-SVM, the most commonly used one, as an example to provide a description.

### 5.1. Least Square Support Vector Machine (LS-SVM)

The LS-SVM, which was a modified SVM algorithm, was proposed by Suykens *et al.* [113] and used to build the nonlinear model. Here, we only briefly describe the main idea of LS-SVM for function estimation. In principle, LS-SVM always fits a linear relation (*y = wx + b*) between the regressors (*x*) and the dependent variable (*y*). The best relation can be obtained by minimizing the cost function (*Q*) containing a penalized regression error term:

(8) $$Q_{\text{LS}-\;\text{SVM}}=\frac{1}{2}w^{T}w+\gamma \sum_{k=1}^{N}e_{k}^{2}$$

subject to:

(9) $$y_{k}=w^{T}\phi (x_{k})+b+e_{k},\;k=1,\;\;\ldots \;\;,\;\;N$$

where *ϕ* : *R^n^* → *R^m^* is the feature map mapping the input space to a usually high-dimensional feature space, *γ* is the relative weight of the error term, and *e_k_* is error variables taking noisy data into accurate and avoiding poor generalization. LS-SVM considers this optimization problem to be a constrained optimization problem and uses a language function to solve it. By solving the Lagrange style of equation (8), the weight coefficients (*w*) can be written as:

(10) $$w=\sum_{k=1}^{N}\alpha_{k}x_{k}^{T}x+b\;\;\text{with}\;\alpha_{k}=2\gamma e_{k}$$

By substituting it into the original regression line (*y* □ *wx + b*), the following result can be obtained:

(11) $$y=\sum_{k=1}^{N}\alpha_{k}x_{k}^{T}x+b$$

It can be seen that the Lagrange multipliers can be defined as:

$$\alpha_{k}=(x_{k}^{T}x+{\left(2\gamma \right)}^{-1}(y_{k}-b))$$

Finding these Lagrange multipliers is very simple, as opposed to with the SVM approach, in which a more difficult relation has to be solved to obtain these values. In addition, it easily allows for a nonlinear regression as an extension of the linear approach by introducing the kernel function. This leads to the following nonlinear regression function:

(12) $$f(x)=\sum_{k=1}^{N}\alpha_{k}K(x,x_{k})+b$$

where *K*(*x, x_k_*) is the kernel function. The value is equal to the inner product of two vectors *x* and *x_k_* in the feature space Φ(*x*) and Φ(*x_k_*); that is, *K*(*x, x_k_*)) = Φ(*x*)^T^Φ(*x_k_*). The choices of a kernel and its specific parameters together with γ have to be tuned by the user. The radial basis function (RBF) kernel *K*(*x, x_k_*) = exp(-‖*x_k_* *– x*‖^2^/σ^2^) is commonly used, and then leave-one-out (LOO) cross-validation is employed to tune the optimized values of the two parameters γ and σ. LS-SVM is a simplification of traditional support vector machine (SVM). It encompasses similar advantages with SVM and its own additional advantages. It only requires solving a set of linear equations (linear programming), which is much easier and computationally simpler than nonlinear equations (quadratic programming) employed by traditional SVM. Therefore, LS-SVM is faster than traditional SVM in treating the same work. The related literature is presented in [37,114–118].

## 6. Gene Expression Programming (GEP)

Gene expression programming was invented by Ferreira in 1999 and was developed from genetic algorithms and genetic programming (GP). GEP uses the same kind of diagram representation of GP, but the entities evolved by GEP (expression trees) are the expression of a genome. GEP is more simple than cellular gene progression. It mainly includes two sides: the chromosomes and the expression trees (ETs). The process of information of gene code and translation is very simple, such as a one-to-one relationship between the symbols of the chromosome and the functions or terminals they represent. The rules of GEP determine the spatial organization of the functions and terminals in the ETs and the type of interaction between sub-ETs. Therefore, the language of the genes and the ETs represents the language of GEP.

### 6.1. The GEP chromosomes, expression trees (ETs), and the mapping mechanism

Each chromosome in GEP is a character string of fixed length, which can be composed of gene from the function set or the terminal set. Using the elements {+, −,*, /, *Q*} as the function set and {*a, b, c, d*} as the terminal set, the following is an example of GEP chromosome of length eight:

$$\left\{\begin{array}{l} 01234567\\ Q*+-abcd \end{array}\right\}$$

where *Q* denotes the square-root function; and *a, b, c, d* are variable (or attribute) names. The above is referred to as Karva notation or *K*-expression. A *K*-expression can be mapped into an ET following a width-first procedure. A branch of the ET stops growing when the last node in this branch is a terminal. The conversion of an ET into a *K*-expression is also very straightforward and can be accomplished by recording the nodes from left to right in each layer of the ET in a top-down fashion to form the string:

(13) $$y=\sqrt{\left(a-b)*(c+d\right)}$$

### 6.2. Description of the GEP algorithm

The purpose of symbolic regression or function finding is to find an expression that can give a good explanation for the dependent variable. The first step is to choose the fitness function. Mathematically, the fitness *f_i_* of an individual program *i* is expressed by the equation:

(14) $$f_{i}=\sum_{j=1}^{n}\left(R-\left|\frac{P_{\left(ij\right)}-T_{j}}{T_{j}}.100\right|\right)$$

where *R* is the selection range, *P_(ij)_* is the value predicted by the individual program *i* for fitness case *j* (out of *n* fitness cases), and *T_j_* is the target value for fitness case *j*. Note that the absolute value term corresponds to the relative error. This term is what is called the precision and if the error is smaller than or is equal to the precision then the error becomes zero. Thus, for a good match the absolute value term is zero and *f_i_* = *f_max_* = *nR*. For some function finding problems it is important to evolve a model that performs well for all fitness cases within a certain relative error (the precision) of the correct value:

(15) $$f_{\left(i,j\right)}=\{\begin{array}{l} 1,E_{\left(i,j\right)}\leq \;p\\ 0, \end{array}$$

where *p* is the precision and *E_(ij)_* is the relative error of an individual program *i* for the fitting case *j*.

The *E_(ij)_* is given by:

(16) $$E_{\left(ij\right)}=\left|\frac{P_{\left(ij\right)}-T_{j}}{T_{j}}•100\right|$$

The second step consists of choosing the set of terminals T and the set of functions F to create the chromosomes. In this problem, the terminal set consists obviously of the independent variable, i.e., *T =* {*a*}. The third step is to choose the chromosomal architecture, i.e., the length of the head and the number of genes. The fourth major step is to choose the linking function. The last major step is to choose the set of genetic operators that cause variation and their rates. These processes are repeated for a pre-specified number of generations until a solution is obtained. In the GEP, the individuals are often selected and copied into the next generation based on their fitness, as determined by roulette-wheel sampling with elitism, which guarantees the survival and cloning of the best individual to the next generation. The variation in the population is introduced by applying one or more genetic operators to select chromosomes, including crossover, mutation, and rotation. The process begins with the random generation of the chromosomes of the initial population. The chromosomes are expressed and the fitness of each individual is evaluated. The individuals are selected according to the fitness to reproduce with modification, leaving progeny with new traits. The individuals of this new generation are, in their turn, subjected to the same developmental process: expression of the genomes, confrontation of the selection environment, and reproduction with modification. To evaluate the ability of the GEP, the correlation coefficient (*R*) was introduced as:

(17) $$C_{i}=\frac{\text{Cov}(T,P)}{\sigma_{\text{t}}\sigma_{p}}$$

where Cov(*T*, *P*) is the covariance of the target and model outputs. *σ_t_* and *σ_p_* are the corresponding standard deviations. GEP is the newest chemometrics method, and Si *et al.* [23,25,19–121] have applied this method to QSAR studies for the first time. The results from their studies are satisfactory and show a promising use in the nonlinear structure-activity/property relationship correlation area, but GEP is congenitally defective as far as reproducibility of the predicted results is concerned, and always deduces too complex equations. This means a higher requirement for a user who is involved with GEP. GEP is now commercialized in the software Automatic Problem Solver 3.0 or GeneXproTools 4.0 [122].

## 7. Project Pursuit Regression (PPR)

PPR, which was developed by Friedman and Stuetzle [123], is a powerful tool for seeking the interesting projections from high-dimensional data into lower dimensional space by means of linear projections. Therefore, it can overcome the curse of dimensionality because it relies on estimation in at most trivariate settings. Friedman and Stuetzle’s concept of PPR avoided many difficulties experienced with other existing nonparametric regression procedures. It does not split the predictor space into two regions, thereby allowing, when necessary, more complex models. In addition, interactions of predictor variables are directly considered because linear combinations of the predictors are modeled with general smooth functions. Another significant property of PPR is that the results of each interaction can be depicted graphically. The graphical output can be used to modify the major parameters of the procedure: the average smoother bandwidth and the terminal threshold. A brief description about PPR is presented here. Given the (*k × n*) data matrix *X*, where *k* is the number of observed variables and *n* is the number of units, and an *m*-dimensional orthonormal matrix *A* (*m × k*), the (*m × n*) matrix *Y* = *AX* represents the coordinates of the projected data onto the m-dimensional (*m < k*) space spanned by the rows of *A*. Because such projections are infinite, it is important to have a technique to pursue a finite sequence of projections that can reveal the most interesting structures of the data. Projection pursuit (PP) is such a powerful tool that combines both ideas of projection and pursuit. In a typical regression problem, PPR aims to approximate the regression pursuit function *f*(*x*) by a finite sum of ridge functions with suitable choices of *α_i_* and *g_i_*:

(18) $$g^{\left(p\right)}(x)=\sum_{i=1}^{p}g_{i}(\alpha_{i}^{T}x)$$

where *α_i_* values are *m × n* orthonormal matrices and *p* is the number of ridge functions. In recent QSAR/QSPR studies [21,37,124–129] PPR was employed as a regression method and always resulted in the best final models. This indicates that PPR is a promising regression method in QSAR/QSPR studies, especially when the correlation between descriptors and activities or properties is nonlinear.

## 8. Local Lazy Regression (LLR)

Most QSAR/QSPR models often capture the global structure-activity/property trends which are present in a whole dataset. In many cases, there may be groups of molecules which exhibit a specific set of features which relate to their activity or property. Such a major feature can be said to represent a local structure activity/property relationship. Traditional models may not recognize such local relationships. LLR is an excellent approach which extracts a prediction by locally interpolating the neighboring examples of the query which are considered relevant according to a distance measure, rather than considering the whole dataset. That will cause the basic core of this approach which is a simple assumption that similar compounds have similar activities or properties; that is, the activities or properties of molecules will change concurrently with the changes in the chemical structure. For one or more query points, “lazy” estimates the value of an unknown multivariate function on the basis of a set of possibly noisy samples of the function itself. Each sample is an input/output pair where the input is a vector and the output is a number. For each query point, the estimation of the input is obtained by combining different local models. Local models considered for combination by lazy are polynomials of zeroth, first, and second degree that fit a set of samples in the neighborhood of the query point. The neighbors are selected according to either the “Manhattan” or the “Euclidean” distance. It is possible to assign weights to the different directions of the input domain for modifying their importance in computation of the distance. The number of neighbors used for identifying local models is automatically adjusted on a query-by-query basis through a leave-one-out validation of models, each fitting a different number of neighbors. The local models are identified by using the recursive least-squares algorithm, and the leave-one-out cross-validation is obtained through the PRESS statistic. We assumed that there existed a small region around *x* which is linear between the dependent variable and the predictor variables. Then we determined the points around *X_NN(x)_* and build a regression model with only the points in *NN_(x)_* using the least-squares method and minimized the squared residuals for the region using this model. So we do not need to build multiple models for each point in the training set beforehand. In essence, when faced with a query point, the approach builds a representative predictive model. Hence, this approach is termed local LLR, which is described below:

(19) $$\beta_{x}={\left(X_{NN(X)}^{T}X_{NN(x)}\right)}^{-1}X_{NN(X)}^{T}Y_{NN(x)}$$

where *X_NN(x)_* was a matrix of independent variables, *Y_NN(x)_* the column vectors representing the dependent variables, for the molecules in the neighborhood of the query point. *β_x_* was the column vectors of regression coefficients. One of the main advantages of LLR is the fact that no *a priori* model needs to be built. This makes it suitable for large data sets, where using all of the observations can normally be time-consuming and even lead to over-fitting. At the same time, because it builds a regression model for each query point, one cannot extract meaningful structure-activity trends for the data set as a whole. That is, the focus of LLR is on predictive ability, rather than interpretability. Like every method, the lazy approach has a number of shortcomings. First, as all of the computations are done at query time, the determination of the local neighborhood must be efficient. Second, uncorrelated features might result in errors in the identification of near neighbors. Finally, it is nontrivial to integrate feature selection in this framework. LLR is generally used to develop linear models for data sets in which the global structure-activity/property relationship is nonlinear in nature. However, as a new arising method in the field of QSAR/QSPR, LLR is not used extensively, with only a few relevant studies shown in references [125,130–132]. It is expected that more application studies involving LLR will appear in future QSAR/QSPR analyses.

## 9. Conclusions

In this paper, we focus on the current mathematical methods used as regression tools in recent QSAR/QSPR studies. Mathematical regression methods are so important for the QSAR/QSPR modeling that the choice of the regression method, most of the time, will determine if the resulted model will be successful or not. Fortunately, more and more new methods and algorithms have been applied to the studies of QSAR/QSPR, including linear and nonlinear, statistics and machine learning. At the same time, the existing methods have been improved. However, it is still a challenge for the researchers to choose suitable methods for modeling their systems. This paper may give some help on the knowledge of these methods, but more practical applications are needed so as to get a thorough understanding and then perform a better application.

## References

[1] Katritzky AR Lobanov VS Karelson M Comprehensive Descriptors for Structural and Statistical Analysis (CODESSA) Ref. Man. Version 2.7.10, 2007

[2] Du H, Wang J, Hu Z, Yao X (2008). Quantitative Structure-Retention relationship study of the constituents of saffron aroma in SPME-GC-MS based on the projection pursuit regression method. Talanta.

[3] Du H, Watzl J, Wang J, Zhang X, Yao X, Hu Z (2008). Prediction of retention indices of drugs based on immobilized artificial membrane chromatography using Projection Pursuit Regression and Local Lazy Regression. J. Sep. Sci.

[4] Du H, Zhang X, Wang J, Yao X, Hu Z (2008). Novel approaches to predict the retention of histidine-containing peptides in immobilized metal-affinity chromatography. Proteomics.

[5] Katritzky AR, Pacureanu L, Dobchev D, Karelson M (2007). QSPR modeling of hyperpolarizabilities. J. Mol. Model.

[6] Ren Y, Liu H, Yao X, Liu M (2007). An accurate QSRR model for the prediction of the GCxGCTOFMS retention time of polychlorinated biphenyl (PCB) congeners. Anal. Bioanal. Chem.

[7] Srivani P, Srinivas E, Raghu R, Sastry GN (2007). Molecular modeling studies of pyridopurinone derivatives--potential phosphodiesterase 5 inhibitors. J. Mol. Graph. Model.

[8] Kahn I, Sild S, Maran U (2007). Modeling the toxicity of chemicals to Tetrahymena pyriformis using heuristic multilinear regression and heuristic back-propagation neural networks. J. Chem. Inf. Model.

[9] Semichem Home Page. http://www.semichem.com/codessa.

[10] Codessa Pro Home Page. http://www.codessa-pro.com/.

[11] Xia B, Liu K, Gong Z, Zheng B, Zhang X, Fan B (2009). Rapid toxicity prediction of organic chemicals to Chlorella vulgaris using quantitative structure-activity relationships methods. Ecotoxicol. Environ. Saf.

[12] Yuan Y, Zhang R, Hu R, Ruan X (2009). Prediction of CCR5 receptor binding affinity of substituted 1-(3,3-diphenylpropyl)-piperidinyl amides and ureas based on the heuristic method, support vector machine and projection pursuit regression. Eur. J. Med. Chem.

[13] Lu WJ, Chen YL, Ma WP, Zhang XY, Luan F, Liu MC, Chen XG, Hu ZD (2008). QSAR study of neuraminidase inhibitors based on heuristic method and radial basis function network. Eur. J. Med. Chem.

[14] Xia B, Ma W, Zheng B, Zhang X, Fan B (2008). Quantitative structure-activity relationship studies of a series of non-benzodiazepine structural ligands binding to benzodiazepine receptor. Eur. J. Med. Chem.

[15] Zhao C, Zhang H, Luan F, Zhang R, Liu M, Hu Z, Fan B (2007). QSAR method for prediction of protein-peptide binding affinity: application to MHC class I molecule HLA-A*0201. J. Mol. Graph. Model.

[16] Rebehmed J, Barbault F, Teixeira C, Maurel F (2008). 2D and 3D QSAR studies of diarylpyrimidine HIV-1 reverse transcriptase inhibitors. J. Comput. Aided Mol. Des.

[17] Agrafiotis DK, Gibbs AC, Zhu F, Izrailev S, Martin E (2007). Conformational sampling of bioactive molecules: a comparative study. J. Chem. Inf. Model.

[18] Si HZ, Wang T, Zhang KJ, Hu ZD, Fan BT (2006). QSAR study of 1,4-dihydropyridine calcium channel antagonists based on gene expression programming. Bioorg. Med. Chem.

[19] Li X, Luan F, Si H, Hu Z, Liu M (2007). Prediction of retention times for a large set of pesticides or toxicants based on support vector machine and the heuristic method. Toxicol. Lett.

[20] Gong ZG, Zhang RS, Xia BB, Hu RJ, Fan BT (2008). Study of nematic transition temperatures in themotropic liquid crystal using heuristic method and radial basis function neural networks and support vector machine. QSAR Comb.Sci.

[21] Yuan YN, Zhang RS, Hu RJ, Ruan XF (2009). Prediction of CCR5 receptor binding affinity of substituted 1-(3,3-diphenylpropyl)-piperidinyl amides and ureas based on the heuristic method, support vector machine and projection pursuit regression. Eur. J. Med. Chem.

[22] Xia BB, Liu KP, Gong ZG, Zheng B, Zhang XY, Fan BT (2009). Rapid toxicity prediction of organic chemicals to Chlorella vulgaris using quantitative structure-activity relationships methods. Ecotoxicol. Environ. Saf.

[23] Luan F, Si HZ, Liu HT, Wen YY, Zhang XY (2008). Prediction of atmospheric degradation data for POPs by gene expression programming. SAR QSAR Environ. Res.

[24] Xia BB, Ma WP, Zheng B, Zhang XY, Fan BT (2008). Quantitative structure-activity relationship studies of a series of non-benzodiazepine structural ligands binding to benzodiazepine receptor. Eur. J. Med. Chem.

[25] Wang T, Si HZ, Chen PP, Zhang KJ, Yao XJ (2008). QSAR models for the dermal penetration of polycyclic aromatic hydrocarbons based on Gene Expression Programming. QSAR Comb. Sci.

[26] Liu KP, Xia BB, Ma WP, Zheng B, Zhang XY, Fan BT (2008). Quantitative structure-activity relationship modeling of triaminotriazine drugs based on Heuristic Method. QSAR Comb. Sci.

[27] Lu WJ, Chen YL, Ma WP, Zhang XY, Luan F, Liu MC, Chen XG, Hu ZD (2008). QSAR study of neuraminidase inhibitors based on heuristic method and radial basis function network. Eur. J. Med. Chem.

[28] Zhao CY, Zhang HX, Luan F, Zhang RS, Liu MC, Hu ZD, Fan BT (2007). QSAR method for prediction of protein-peptide binding affinity: Application to MHC class I molecule HLA-A*0201. J. Mol. Graph. Model.

[29] Li HZ, Liu HX, Yao XJ, Liu MC, Hu ZD, Fan BT (2007). Quantitative structure-activity relationship study of acyl ureas as inhibitors of human liver glycogen phosphorylase using least squares support vector machines. Chemometr. Intel. Lab. Syst.

[30] Qin S, Liu HX, Wang J, Yao XJ, Liu MC, Hu ZD, Fan BT (2007). Quantitative Structure-Activity Relationship study on a series of novel ligands binding to central benzodiazepine receptor by using the combination of Heuristic Method and Support Vector Machines. QSAR Comb. Sci.

[31] Ma WP, Luan F, Zhao CY, Zhang XY, Liu MC, Hu ZD, Fan BT (2006). QSAR prediction of the penetration of drugs across a polydimethylsiloxane membrane. QSAR Comb. Sci.

[32] Luan F, Ma WP, Zhang XY, Zhang HX, Liu MC, Hu ZD, Fan BT (2006). Quantitative structure-activity relationship models for prediction of sensory irritants (logRD(50)) of volatile organic chemicals. Chemosphere.

[33] Si HZ, Yao XJ, Liu HX, Wang J, Li JZ, Hu ZD, Liu MC (2006). Prediction of binding rate of drug to human plasma protein based on heuristic method and support vector machine. Acta Chim. Sinica.

[34] Luan F, Ma WP, Zhang XY, Zhang HX, Liu MC, Hu ZD, Fan BT (2006). QSAR study of polychlorinated dibenzodioxins, dibenzofurans, and Biphenyls using the heuristic method and support vector machine. QSAR Comb. Sci.

[35] Gharagheizi F, Tirandazi B, Barzin R (2009). Estimation of aniline point temperature of pure hydrocarbons: A quantitative structure-property relationship approach. Ind. Eng. Chem. Res.

[36] Riahi S, Mousavi MF, Ganjali MR, Norouzi P (2008). Application of correlation ranking procedure and artificial neural networks in the modeling of liquid chromatographic retention times (tR) of various pesticides. Anal. Lett.

[37] Du HY, Wang J, Hu ZD, Yao XJ, Zhang XY (2008). Prediction of fungicidal activities of rice blast disease based on least-squares support vector machines and project pursuit regression. J. Agric. Food Chem.

[38] Gharagheizi F, Mehrpooya M (2008). Prediction of some important physical properties of sulfur compounds using quantitative structure-properties relationships. Mol. Div.

[39] Sattari M, Gharagheizi F (2008). Prediction of molecular diffusivity of pure components into air: A QSPR approach. Chemosphere.

[40] Gharagheizi F, Alamdari RF (2008). Prediction of flash point temperature of pure components using a Quantitative Structure-Property Relationship model. QSAR Comb. Sci.

[41] Gharagheizi F, Fazeli A (2008). Prediction of the Watson characterization factor of hydrocarbon components from molecular properties. QSAR Comb. Sci.

[42] Om AS, Ryu JC, Kim JH (2008). Quantitative structure-activity relationships for radical scavenging activities of flavonoid compounds by GA-MLR technique. Mol. Cell. Toxicol.

[43] Riahi S, Ganjali MR, Pourbasheer E, Norouzi P (2008). QSRR study of GC retention indices of essential-oil compounds by multiple linear regression with a genetic algorithm. Chromatographia.

[44] Hashemianzadeh M, Safarpour MA, Gholamjani-Moghaddam K, Mehdipour AR (2008). DFT-based QSAR study of valproic acid and its derivatives. QSAR Comb. Sci.

[45] Gharagheizi F (2008). A new molecular-based model for prediction of enthalpy of sublimation of pure components. Thermochim. Acta.

[46] Gharagheizi F (2008). QSPR studies for solubility parameter by means of Genetic Algorithm-Based Multivariate Linear Regression and generalized regression neural network. QSAR Comb. Sci.

[47] Gharagheizi F, Alamdari RF (2008). A molecular-based model for prediction of solubility of C-60 fullerene in various solvents. Fuller. Nanotub. Carbon Nanostr.

[48] Carlucci G, D'Archivio AA, Maggi MA, Mazzeo P, Ruggieri F (2007). Investigation of retention behaviour of non-steroidal anti-inflammatory drugs in high-performance liquid chromatography by using quantitative structure-retention relationships. Anal. Chim. Acta.

[49] Gharagheizi F A new accurate neural network quantitative structure-property relationship for prediction of theta (lower critical solution temperature) of polymer solutions E-Polymers 2007, No. 114

[50] Elliott GN, Worgan H, Broadhurst D, Draper J, Scullion J (2007). Soil differentiation using fingerprint Fourier transform infrared spectroscopy, chemometrics and genetic algorithm-based feature selection. Soil Biol. Biochem.

[51] Gharagheizi F (2007). QSPR analysis for intrinsic viscosity of polymer solutions by means of GA-MLR and RBFNN. Comput. Mater. Sci.

[52] Deeb O, Hemmateenejad B, Jaber A, Garduno-Juarez R, Miri R (2007). Effect of the electronic and physicochemical parameters on the carcinogenesis activity of some sulfa drugs using QSAR analysis based on genetic-MLR and genetic-PLS. Chemosphere.

[53] Vatani A, Mehrpooya M, Gharagheizi F (2007). Prediction of standard enthalpy of formation by a QSPR model. Int. J. Mol. Sci.

[54] Jung M, Tak J, Lee Y, Jung Y (2007). Quantitative structure-activity relationship (QSAR) of tacrine derivatives against acetylcholinesterase (ACNE) activity using variable selections. Bioorg. Med. Chem. Lett.

[55] Fisz JJ (2006). Combined genetic algorithm and multiple linear regression (GA-MLR) optimizer: Application to multi-exponential fluorescence decay surface. J. Phys. Chem. A.

[56] Word H (1966). Research Papers in Statistics.

[57] Jores-Kong H, Word H (1982). Systems under Indirect Observation: Causality, structure, prediction.

[58] Rogers D, Hopfinger AJ (1994). Application of genetic function approximation to quantitative structure-activity-relationships and quantitative structure-property relationships. J. Chem. Inf. Comput. Sci.

[59] Fan Y, Shi LM, Kohn KW, Pommier Y, Weinstein JN (2001). Quantitative structure-antitumor activity relationships of camptothecin analogues: Cluster analysis and genetic algorithm-based studies. J. Med. Chem.

[60] Sammi T, Silakari O, Ravikumar M (2009). Three-dimensional quantitative structure-activity relationship (3D-QSAR) studies of various benzodiazepine analogues of gamma-secretase inhibitors. J. Mol. Model.

[61] Li ZG, Chen KX, Xie HY, Gao JR (2009). Quantitative structure - activity relationship analysis of some thiourea derivatives with activities against HIV-1 (IIIB). QSAR Comb. Sci.

[62] Samee W, Nunthanavanit P, Ungwitayatorn J (2008). 3D-QSAR investigation of synthetic antioxidant chromone derivatives by molecular field analysis. Int. J. Mol. Sci.

[63] Nunthanavanit P, Anthony NG, Johnston BF, Mackay SP, Ungwitayatorn J (2008). 3D-QSAR studies on chromone derivatives as HIV-1 protease inhibitors: Application of molecular field analysis. Arch. Der. Pharm.

[64] Kansal N, Silakari O, Ravikumar M (2008). 3D-QSAR studies of various diaryl urea derivatives of multi-targeted receptor tyrosine kinase inhibitors: Molecular field analysis approach. Lett. Drug Des. Dis.

[65] Joseph TB, Kumar B, Santhosh B, Kriti S, Pramod AB, Ravikumar M, Kishore M (2008). Quantitative structure activity relationship and pharmacophore studies of adenosine receptor A(2B) inhibitors. Chem. Biol. Drug Des.

[66] Equbal T, Silakari O, Ravikumar M (2008). Exploring three-dimensional quantitative structural activity relationship (3D-QSAR) analysis of SCH 66336 (Sarasar) analogues of farnesyltransferase inhibitors. Eur. J. Med. Chem.

[67] Bhonsle JB, Bhattacharjee AK, Gupta RK (2007). Novel semi-automated methodology for developing highly predictive QSAR models: application for development of QSAR models for insect repellent amides. J. Mol. Model.

[68] Thomas Leonard J, Roy K (2006). Comparative QSAR modeling of CCR5 receptor binding affinity of substituted 1-(3,3-diphenylpropyl)-piperidinyl amides and ureas. Bioorg. Med. Chem. Lett.

[69] Roy K, Leonard JT (2006). Topological QSAR modeling of cytotoxicity data of anti-HIV 5-phenyl-1-phenylamino-imidazole derivatives using GFA, G/PLS, FA and PCRA techniques. Indian J. Chem. Sect. A-Inorg. Bio-Inorg. Phys. Theor. Anal. Chem.

[70] Davies MN, Hattotuwagama CK, Moss DS, Drew MGB, Flower DR (2006). Statistical deconvolution of enthalpic energetic contributions to MHC-peptide binding affinity. BMC Struct Biol.

[71] Mandal AS, Roy K (2009). Predictive QSAR modeling of HIV reverse transcriptase inhibitor TIBO derivatives. Eur. J. Med. Chem.

[72] Leonard JT, Roy K (2008). Exploring molecular shape analysis of styrylquinoline derivatives as HIV-1 integrase inhibitors. Eur. J. Med. Chem.

[73] Roy K, Ghosh G (2006). QSTR with extended topochemical atom (ETA) indices 8.(a) QSAR for the inhibition of substituted phenols on germination rate of Cucumis sativus using chemometric tools. QSAR Comb. Sci.

[74] Leonard JT, Roy K (2006). Comparative QSAR modeling of CCR5 receptor binding affinity of substituted 1-(3,3-diphenylpropyl)-piperidinyl amides and ureas. Bioorg. Med. Chem. Lett.

[75] Wold S, Antti H, Lindgren F, Ohman J (1998). Orthogonal signal correction of near-infrared spectra. Chemometr. Intel. Lab. Syst.

[76] Yin PY, Mohemaiti P, Chen J, Zhao XJ, Lu X, Yimiti A, Upur H, Xu GW (2008). Serum metabolic profiling of abnormal savda by liquid chromatography/mass spectrometry. J. Chromatogr. B-Anal. Technol. Biomed. Life Sci.

[77] Samadi-Maybodi A, Darzi S (2008). Simultaneous determination of vitamin B12 and its derivatives using some of multivariate calibration 1 (MVC1) techniques. Spectrochim. Acta A-Mol. Biomol. Spectrosc.

[78] Niazi A, Jafarian B, Ghasemi J (2008). Kinetic spectrophotometric determination of trace amounts of palladium by whole kinetic curve and a fixed time method using resazurine sulfide reaction. Spectrochim. Acta A-Mol. Biomol. Spectrosc.

[79] Niazi A, Goodarzi M (2008). Orthogonal signal correction-partial least squares method for simultaneous spectrophotometric determination of cypermethrin and tetramethrin. Spectrochim. Acta A-Mol. Biomol. Spectrosc.

[80] Niazi A, Amjadi E, Nori-Shargh D, Bozorghi SJ (2008). Simultaneous voltammetric determination of lead and tin by adsorptive differential pulse stripping method and orthogonal signal correction-partial least squares in water samples. J. Chinese Chem. Soc.

[81] Karimi MA, Ardakani MM, Behjatmanesh-Ardakani R, Nezhad MRH, Amiryan H (2008). Individual and simultaneous determinations of phenothiazine drugs using PCR, PLS and (OSC)-PLS multivariate calibration methods. J. Serb. Chem. Soc.

[82] Cho HW, Kim SB, Jeong MK, Park Y, Miller NG, Ziegler TR, Jones DP (2008). Discovery of metabolite features for the modelling and analysis of high-resolution NMR spectra. Int. J. Data Min. Bioinf.

[83] Cheng Z, Zhu AS, Zhang LQ (2008). Quantitative analysis of electronic absorption spectroscopy by piecewise orthogonal signal correction and partial least square. Guang Pu Xue Yu Guang Pu Fen Xi.

[84] Cheng Z, Zhu AS, Zhang LQ (2008). Quantitative analysis of electronic absorption spectroscopy by piecewise orthogonal signal correction and partial least square. Spectrosc. Spectr. Anal.

[85] Cheng Z, Zhu AS (2008). Piecewise orthogonal signal correction approach and its application in the analysis of wheat near-infrared spectroscopic data. Chinese J. Anal. Chem.

[86] Rouhollahi A, Rajabzadeh R, Ghasemi J (2007). Simultaneous determination of dopamine and ascorbic acid by linear sweep voltammetry along with chemometrics using a glassy carbon electrode. Microchim. Acta.

[87] Psihogios NG, Kalaitzidis RG, Dimou S, Seferiadis KI, Siamopoulos KC, Bairaktari ET (2007). Evaluation of tubulointerstitial lesions' severity in patients with glomerulonephritides: An NMR-Based metabonomic study. J. Proteome Res.

[88] Niazi A, Azizi A, Leardi R (2007). A comparative study between PLS and OSC-PLS in the simultaneous determination of lead and mercury in water samples: effect of wavelength selection. Can. J. Anal. Sci. Spectrosc.

[89] Priolo N, Arribere CM, Caffini N, Barberis S, Vazquez RN, Luco JM (2001). Isolation and purification of cysteine peptidases from the latex of Araujia hortorum fruits - Study of their esterase activities using partial least-squares (PLS) modeling. J. Mol. Catal. B-Enzym.

[90] Yang SB, Xia ZN, Shu M, Mei H, Lue FL, Zhang M, Wu YQ, Li ZL (2008). VHSEH Descriptors for the Development of QSAMs of Peptides. Chem. J. Chinese Univ.

[91] Liang GZ, Mei H, Zhou Y, Yang SB, Wu SR, Li ZL (2006). Using SZOTT descriptors for the development of QSAMs of peptides. Chem. J. Chinese Univ.

[92] Zhao CY, Boriani E, Chana A, Roncaglioni A, Benfenati E (2008). A new hybrid system of QSAR models for predicting bioconcentration factors (BCF). Chemosphere.

[93] Qi J, Niu JF, Wang LL (2008). Research on QSPR for n-octanol-water partition coefficients of organic compounds based on genetic algorithms-support vector machine and genetic algorithms-radial basis function neural networks. Huanjing Kexue.

[94] Luan F, Liu HT, Wen YY, Zhang XY (2008). Prediction of quantitative calibration factors of some organic compounds in gas chromatography. Analyst.

[95] Luan F, Liu HT, Wen YY, Zhang XY (2008). Quantitative structure-property relationship study for estimation of quantitative calibration factors of some organic compounds in gas chromatography. Anal. Chim. Acta.

[96] Luan F, Liu HT, Ma WP, Fan BT (2008). QSPR analysis of air-to-blood distribution of volatile organic compounds. Ecotoxicol. Environ. Saf.

[97] Chen HF (2008). Quantitative predictions of gas chromatography retention indexes with support vector machines, radial basis neural networks and multiple linear regression. Anal. Chim. Acta.

[98] Zhao CY, Zhang HX, Zhang XY, Liu MC, Hu ZD, Fan BT (2006). Application of support vector machine (SVM) for prediction toxic activity of different data sets. Toxicology.

[99] Tetko IV, Solov’ev VP, Antonov AV, Yao XJ, Doucet JP, Fan BT, Hoonakker F, Fourches D, Jost P, Lachiche N, Varnek A (2006). Benchmarking of linear and nonlinear approaches for quantitative structure-property relationship studies of metal complexation with ionophores. J. Chem. Inf. Model.

[100] Shi J, Luan F, Zhang HX, Liu MC, Guo QX, Hu ZD, Fan BT (2006). QSPR study of fluorescence wavelengths (lambda(ex)/lambda(em)) based on the heuristic method and radial basis function neural networks. QSAR Comb. Sci.

[101] Ma WP, Luan F, Zhang HX, Zhang XY, Liu MC, Hu ZD, Fan BT (2006). Accurate quantitative structure-property relationship model of mobilities of peptides in capillary zone electrophoresis. Analyst.

[102] Luan F, Zhang X, Zhang H, Zhang R, Liu M, Hu Z, Fan B (2006). Prediction of standard Gibbs energies of the transfer of peptide anions from aqueous solution to nitrobenzene based on support vector machine and the heuristic method. J. Comput. Aided Mol. Des.

[103] Specht DF (1991). A general regression neural network. IEEE Trans. Neur. Netw.

[104] Szaleniec M, Tadeusiewicz R, Witko M (2008). How to select an optimal neural model of chemical reactivity?. Neurocomputing.

[105] Ji L, Wang XD, Luo S, Qin LA, Yang XS, Liu SS, Wang LS (2008). QSAR study on estrogenic activity of structurally diverse compounds using generalized regression neural network. Sci. China Ser. B-Chem.

[106] Mager PP (2007). Subset selection and docking of human P2X7 inhibitors. Curr. Comput. Aided Drug Des.

[107] Ibric S, Jovanovic M, Djuric Z, Parojcic J, Solomun L, Lucic B (2007). Generalized regression neural networks in prediction of drug stability. J. Pharm. Pharmacol.

[108] Yap CW, Li ZR, Chen YZ (2006). Quantitative structure-pharmacokinetic relationships for drug clearance by using statistical learning methods. J. Mol. Graph. Model.

[109] Agatonovic-Kustrin S, Turner JV (2006). Artificial neural network modeling of phytoestrogen binding to estrogen receptors. Lett. Drug Des. Disc.

[110] Cortes C, Vapnik V (1995). Support-Vector Networks. Mach. Learn.

[111] Vapnik V (1999). The Support Vector method of function estimation. US Patent 5,950,146.

[112] Wang WJ Xu ZB Lu WZ Zhang XY Determination of the spread parameter in the Gaussian kernel for classification and regression Neurocomputing 2003 55, PII S0925-2312(02)00632-X.

[113] Suykens JAK, Vandewalle J (1999). Least squares support vector machine classifiers. Neural Process. Lett.

[114] Yuan YN, Zhang RS, Hu RJ, Ruan XF (2008). Prediction of volatile components retention time in blackstrap molasses by least-squares support vector machine. QSAR Comb. Sci.

[115] Niazi A, Jameh-Bozorghi S, Nori-Shargh D (2008). Prediction of toxicity of nitrobenzenes using ab initio and least squares support vector machines. J. Hazard. Mater.

[116] Goudarzi N Goodarzi M Prediction of the logarithmic of partition coefficients (log P) of some organic compounds by least square-support vector machine (LS-SVM) Mol Phys 2008 106, DOI 10.1080/00268970802577834|PII 905993443.

[117] Liu H, Papa E, Walker JD, Gramatica P (2007). In silico screening of estrogen-like chemicals based on different nonlinear classification models. J. Mol. Graph. Model.

[118] Liu HX, Yao XJ, Zhang RS, Liu MC, Hu ZD, Fan BT (2005). Prediction of the tissue/blood partition coefficients of organic compounds based on the molecular structure using least-squares support vector machines. J. Comput. Aided Mol. Des.

[119] Si HZ, Wang T, Zhang KJ, Duan YB, Yuan SP, Fu AP, Hu ZD (2007). Quantitative structure activity relationship model for predicting the depletion percentage of skin allergic chemical substances of glutathione. Anal. Chim. Acta.

[120] Si HZ, Zhang KJ, Hu ZD, Fan BT (2007). QSAR model for prediction capacity factor of molecular imprinting polymer based on gene expression programming. QSAR Comb. Sci.

[121] Si HZ, Yuan SP, Zhang KJ, Fu AP, Duan YB, Hue ZD (2008). Quantitative structure activity relationship study on EC5.0 of anti-HIV drugs. Chemometr. Intel. Lab. Syst.

[122] Gepsoft Home Page. http://www.gepsoft.com/.

[123] Friedman JH, Stuetzle W (1981). Projection Pursuit Regression. J. Am. Stat. Assoc.

[124] Yuan YN, Zhang RS, Hu RJ (2009). Prediction of Photolysis of PCDD/Fs Adsorbed to Spruce [Picea abies (L.) Karst.] Needle Surfaces Under Sunlight Irradiation Based on Projection Pursuit Regression. QSAR Comb. Sci.

[125] Du HY, Zhang XY, Wang X, Yao XJ, Hu ZD (2008). Novel approaches to predict the retention of histidine-containing peptides in immobilized metal-affinity chromatography. Proteomics.

[126] Du HY, Wang J, Zhang XY, Hu ZD (2008). A novel quantitative structure-activity relationship method to predict the affinities of MT3 melatonin binding site. Eur. J. Med. Chem.

[127] Du HY, Wang J, Watzl J, Zhang XY, Hu ZD (2008). Prediction of inhibition of matrix metalloproteinase inhibitors based on the combination of Projection Pursuit Regression and Grid Search method. Chemometr. Intel. Lab. Syst.

[128] Ren YY, Liu HX, Yao XJ, Liu MC (2007). Prediction of ozone tropospheric degradation rate constants by projection pursuit regression. Anal. Chim. Acta.

[129] Ren YY, Liu HX, Li SY, Yao XJ, Liu MC (2007). Prediction of binding affinities to beta(1) isoform of human thyroid hormone receptor by genetic algorithm and projection pursuit regression. Bioorg. Med. Chem. Lett.

[130] Gunturi SB, Archana K, Khandelwal A, Narayanan R (2008). Prediction of hERG Potassium Channel Blockade Using kNN-QSAR and Local Lazy Regression Methods. QSAR Comb. Sci.

[131] Du HY, Watzl J, Wang J, Zhang XY, Yaol XJ, Hu ZD (2008). Prediction of retention indices of drugs based on immobilized artificial membrane chromatography using Projection Pursuit Regression and Local Lazy Regression. J. Sep. Sci.

[132] Guha R, Dutta D, Jurs PC, Chen T (2006). Local lazy regression: Making use of the neighborhood to improve QSAR predictions. J. Chem. Inf. Model.

